# Neutralizing Monoclonal Antibody, mAb 10D8, Is an Effective Detoxicant against Abrin-a Both In Vitro and In Vivo

**DOI:** 10.3390/toxins14030164

**Published:** 2022-02-23

**Authors:** Zhi Li, Hua Xu, Bo Ma, Li Luo, Lei Guo, Pingping Zhang, Yong Zhao, Lili Wang, Jianwei Xie

**Affiliations:** 1 State Key Laboratory of Toxicology and Medical Countermeasures, Laboratory of Toxicant Analysis, Institute of Pharmacology and Toxicology, Academy of Military Medical Sciences, Beijing 100850, China; lz880722@163.com (Z.L.); mbcarl@163.com (B.M.); luolitongxie@163.com (L.L.); guolei@bmi.ac.cn (L.G.); wangll@bmi.ac.cn (L.W.); 2 State Key Laboratory of Pathogen and Biosecurity, Beijing Key Laboratory of POCT for Bioemergency and Clinic, Beijing Institute of Microbiology and Epidemiology, Beijing 100071, China; acedreams@126.com (P.Z.); zhaoyong179@139.com (Y.Z.)

**Keywords:** abrin, monoclonal antibody, neutralizing antibody, antidote, mechanism

## Abstract

Abrin is a types II ribosome-inactivating protein (RIP) isolated from *Abrus precatorious* seeds, which comprises a catalytically active A chain and a lectin-like B chain linked by a disulfide bond. Four isotoxins of abrin have been reported with similar amino-acid composition but different cytotoxicity, of which abrin-a is the most potent toxin. High lethality and easy availability make abrin a potential bioterrorism agent. However, there are no antidotes available for managing abrin poisoning, and treatment is only symptomatic. Currently, neutralizing antibodies remain the most effective therapy against biotoxin poisoning. In this study, we prepared, identified, and acquired a high-affinity neutralizing monoclonal antibody (mAb) 10D8 with a potent pre- and post-exposure protective effect against cytotoxicity and animal toxicity induced by abrin-a or abrin crude extract. The mAb 10D8 could rescue the mouse injected intraperitoneally with a 25 × LD_50_ dose of abrin-a from lethality and prevent tissue damages. Results indicated that 10D8 does not prevent the binding and internalization of abrin-a to cells but inhibits the enzymatic activity of abrin-a and reduces protein synthesis inhibition of cells. The high affinity, good specificity, and potent antitoxic efficiency of 10D8 make it a promising candidate for therapeutic antibodies against abrin.

## 1. Introduction

Abrin is a type II ribosome-inactivating protein (RIP) isolated from *Abrus precatorious* seeds, which inhibits protein synthesis in eukaryotic cells and consequently triggers apoptosis [1,2]. Abrin has similar structure, properties, and functional characteristics to ricin, but it is reported to be more toxic than ricin [3,4]. As known, ricin is one of the most toxic plant toxins with LD_50_ values in mouse of 2–10 µg/kg and an estimated human lethal dose of 5–10 μg/kg body weight, respectively [5,6]. Abrin consists of an enzymatic A chain possessing *N*-glycosidase activity and a galactose-specific B chain responsible for binding and trafficking of the toxin in cells which are linked by a disulfide bond [7]. The USA Center for Disease Control and Prevention (CDC) has classified abrin as a Category B agent. Furthermore, the high lethality, easy availability, and lack of antidote make abrin a potential bioterrorism agent. The estimated human fatal dose of abrin is about 0.1–1 μg/kg body weight [8,9]. Four different isoforms of abrin, named abrin-a, -b, -c, and -d, and an *Abrus* agglutinin (AAG) have been isolated from the seeds of *Abrus precatorius* [10]. These four isoforms have similar amino-acid composition with 78% protein identity but different toxicity, of which abrin-a is the most potent toxin both in vitro and in vivo [11,12,13]. In addition, the content of abrin-a is more than five times higher than the other three isoforms in seeds from Taiwan province, China [11]. Many severe abrin poisoning cases and even death have been reported due to accidental and intentional abrin poisoning through ingestion, inhalation, and injection [14]. Currently, treatment for abrin poisoning is symptomatic, and there are no approved antidotes against abrin intoxications [15]. Neutralizing antibodies are known as a specific and effective strategy against biotoxin poisoning. For abrin, there are rare reports on neutralizing monoclonal antibodies (mAbs) with only a prophylactic effect, a limited post-exposure protective effect in vivo, or an unclear mechanism [16,17,18]. Moreover, the specificity and selectivity of the reported antibodies against abrin isoforms are completely unclear. In this study, we prepared, identified, and acquired a high-affinity neutralizing mAb 10D8 with potent pre- and post-exposure protective effect against abrin-a intoxication. In order to understand the protection mechanism of 10D8, cell and cell-free systems were employed to explore the potential action between the antibody and abrin-a. Results indicated that 10D8 recognizes and binds with the A chain of abrin-a to inhibit the enzymatic activity of abrin-a and reduce protein synthesis inhibition of cells, without preventing the binding and internalizing of abrin-a to cells.

## 2. Results

### 2.1. Production and Screening of the Hybridomas

Hybridomas producing antibodies specific for abrin were successfully established from splenocytes following immunization with inactivated purified abrin-a. Fusion of splenocytes from immunized mice with NS-1 myeloma cells produced more than 100 hybridoma lines, of which 16 cell lines were selected on the basis of their strong reactivity with abrin-a in indirect ELISA. Specific mAbs were purified by using protein G column chromatography, and the affinity was further evaluated by ELISA; finally, five mAbs, i.e., 10D8, 10C9, 5A10, 5G7, and 17C12, were chosen for the subsequent experiments due to their high affinity. All of the mAbs were determined as IgG1 kappa chain isotype by rapid isotyping cassettes represented by 10D8 (Supplementary Material Figure S1, Table S1).

### 2.2. Identification of the Specificity and Cross-Reactivity of mAbs

**Figure 1:**
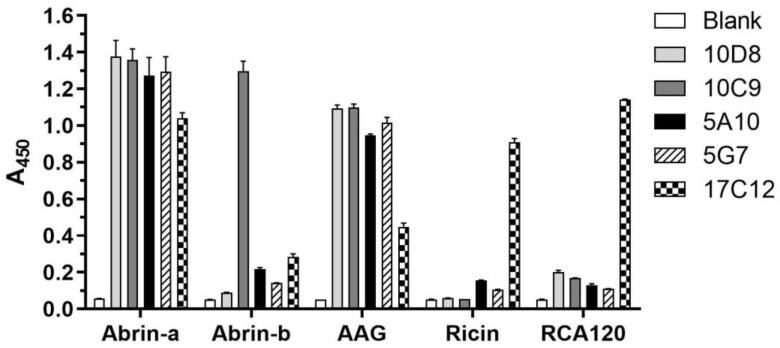
ELISA results demonstrating the specificity and cross-reactivity of monoclonal antibodies. Microplates were coated with abrin-a, abrin-b, AAG, ricin, or RCA120 at 4 °C overnight and blocked with 1% (*m*/*v*) BSA for 2 h at 37 °C. The mAbs were added and incubated for 1 h at 37 °C. Wells were washed with PBST, before adding HRP-labeled anti-mouse antibody and then incubating for 40 min at 37 °C. Wells were washed with PBST, before adding TMB substrate solution and incubating for 10 min. The reaction was terminated by H_2_SO_4_, and the absorbance at 450 nm was measured.

To determine the specificity of the purified mAbs, we performed ELISA. The results demonstrated that all mAbs tested (10D8, 10C9, 5A10, 5G7, 17C12) recognize abrin-a and AAG but not ricin and *Ricinus communis* agglutinin (RCA120), except for 17C12 which showed poor specificity and selectivity. Furthermore, 10C9 also showed binding activity against abrin-b (Figure 1). The binding affinity with abrin-a of these mAbs was further evaluated by surface plasmon resonance (SPR) performed in our previous work, which showed that the K_D_ value of 10D8 is 4.9 ± 0.5 pM, while that of 10C9, 5A10, and 5G7 also approached pM levels (Supplementary Material Table S1) [19]. Taken together, mAb 10D8 showed the best affinity and specificity, along with the least cross-reactivity, to abrin-b and ricin.

### 2.3. Abrin-a Induced Apoptosis Is Inhibited by 10D8

**Figure 2:**
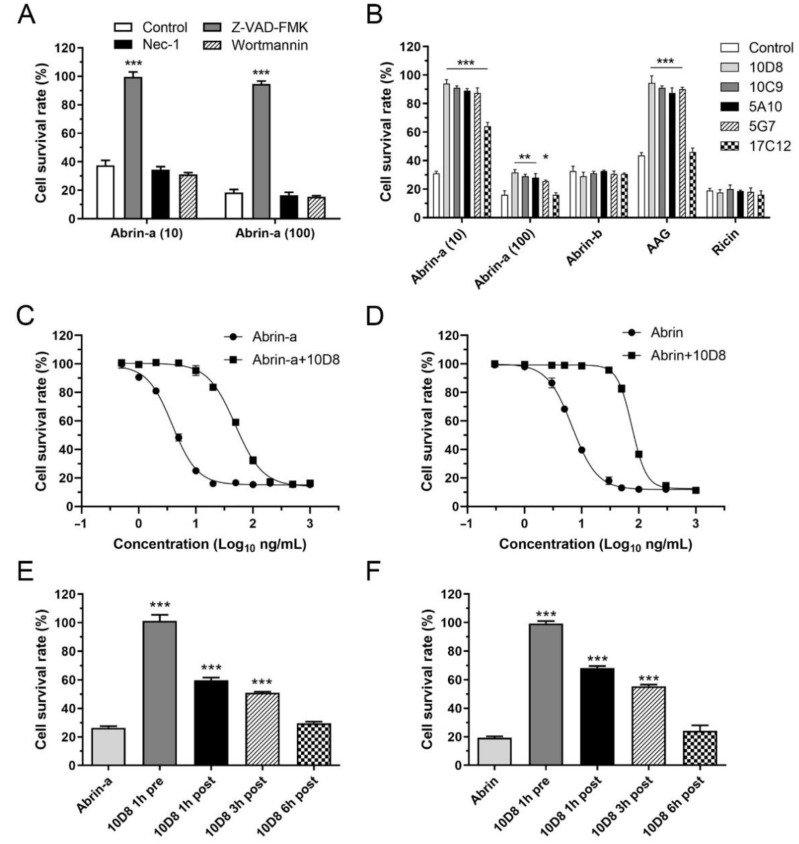
Abrin-a-induced cell apoptosis is inhibited by 10D8. HeLa cells were seeded in 96-well cell culture plates at a density of 1 × 10^4^ cells/well. (**A**) HeLa cells were pretreated with Z-VAD-FMK (50 μM), Nec-1 (50 μM), or wortmannin (100 nM) 1 h prior to abrin-a intoxication. Then, abrin-a (10 or 100 ng/mL) was added to each well. (**B**) Abrin-a (10 or 100 ng/mL), abrin-b (1000 ng/mL), AAG (100 ng/mL), or ricin (100 ng/mL) was preincubated with or without 10 μg/mL monoclonal antibodies at 37 °C for 1 h. (**C**,**D**) Abrin-a or abrin crude extract was diluted with serum-free RPMI-1640 medium at various concentrations and preincubated with or without 10D8 (10 μg/mL) at 37 °C for 1 h. (**E**,**F**) 10D8 (10 μg/mL) was added into cells 1 h before or 1, 3, or 6 h after abrin-a (10 ng/mL) or abrin crude extract (30 ng/mL) intoxication. Cells were incubated at 37 °C for 24 h before adding CCK-8 reagent. Absorbance at 450 nm was measured, and the survival rate of cells was calculated. Data are shown as the mean ± SD, *n* = 3. Statistical significance was determined using one-way ANOVA (* *p* < 0.05, ** *p* < 0.01, *** *p* < 0.001 vs. control (**A**,**B**); *** *p* < 0.001 vs. abrin-a (**E**); *** *p* < 0.001 vs. abrin (**F**)).

It has been reported that abrin induces apoptosis in HeLa cells and three leukemic cell lines through caspase activation [20,21]. To preliminarily explore whether abrin-a intoxication induces cell death other than apoptosis, HeLa cells were treated with abrin-a in the presence or absence of chemical inhibitors of different cell death pathways. Z-VAD-FMK (pan-caspase inhibitor, for apoptosis) but not Nec-1 (RIPK1 inhibitor, for necroptosis) or wortmannin (PI3K inhibitor, for autophagy) treatment significantly abolished abrin-a induced cell death, suggesting that abrin-a induced cell death is mainly apoptosis in HeLa cells, consistent with previous studies (Figure 2A). Preincubation of 10D8, 10C9, 5A10, and 5G7 inhibited abrin-a- and AAG-induced cell death but did not affect abrin-b- and ricin-induced cell death (Figure 2B). To determine the protective efficacy of 10D8 in vitro, cells were treated with different concentrations of abrin-a; the results showed that 10 μg/mL 10D8 has a protective effect against up to 100 ng/mL abrin-a (Figure 2B,C) or abrin crude extract (Figure 2D). Treatment with 10D8 at different timepoints showed that 1 h pre-exposure treatment was the most effective, 1 to 3 h post-exposure treatment inhibited abrin-a- or abrin crude extract-induced cell death, and 6 h post-exposure treatment was ineffective (Figure 2E,F).

### 2.4. 10D8 Inhibits the Enzymatic Activity of Abrin-a A Chain and Reduces Protein Synthesis Inbibition of Cells

**Figure 3:**
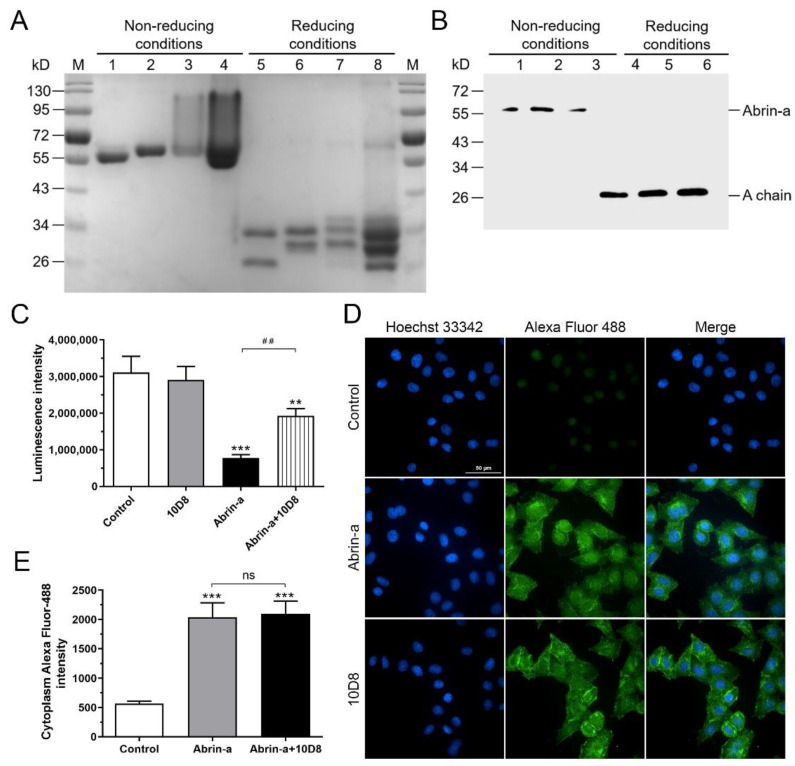
The mAb 10D8 inhibits the enzymatic activity of abrin-a A chain and reduces protein synthesis inhibition of cells. (**A**) Abrin-a, abrin-b, and AAG (each 4 μg) and 20 μg of abrin crude extract were loaded and separated on 10% SDS-PAGE under nonreducing (lanes 1–4) and reducing conditions (lanes 5–8) and stained with Coomassie Blue solution. M: marker; Lane 1, 5: abrin-a; Lane 2, 6: abrin-b; Lane 3, 7: AAG; Lane 4, 8: abrin crude extract. (**B**) Abrin-a (100 ng) was loaded and separated on 10% SDS-PAGE gels under reducing (lanes 1–3) or nonreducing conditions (lanes 4–6), and examined by Western blot assay. The mAb 10D8 was used as the detection antibody. (**C**) Abrin-a (10 ng/mL) alone or along with 10D8 (10 μg/mL) was added to TNT^®^ T7 coupled reticulocyte lysate systems. The extent of the synthesized enzyme luciferase was determined by measuring the luminescence. (**D**,**E**) HeLa cells were seeded in a 96-well black cell culture plate at a density of 1 × 10^4^ cells/well. The cells were treated with abrin-a (100 ng/mL) or abrin-a preincubated with 10D8 (20 μg/mL) for 3 h at 37 °C. The cells were fixed, stained, imaged, and analyzed using a high-content cell analysis system. Scale bar: 50 μm. Data are shown as the mean ± SD, *n* = 3. Statistical significance was determined using one-way ANOVA (** *p* < 0.01, *** *p* < 0.001 vs. control, ns—not significant; ^##^ *p* < 0.01).

SDS-PAGE and Western blot under reducing or nonreducing conditions were performed to explore the epitopes recognized by 10D8. Abrin-a, abrin-b, and AAG displayed different profiles in reducing and nonreducing SDS-PAGE, while the abrin crude extract showed characteristics of all three toxins (Figure 3A). The results demonstrated that 10D8 recognizes and binds the A chain of abrin-a (Figure 3B). The A chain of abrin specifically depurinates 28S rRNA and inhibits protein synthesis. To determine whether 10D8 neutralizes abrin toxicity by inhibiting the enzymatic activity of abrin A chain, a cell-free rabbit reticulocyte lysate system was utilized to measure the enzymatic activity of abrin-a in the absence or presence of 10D8. The results showed that protein synthesis was significantly inhibited by abrin-a, and that 10D8 preincubation reduced protein synthesis inhibition of abrin-a treated cells (Figure 3C).

To examine whether 10D8 inhibits the internalization of abrin into cells, cells treated with abrin-a and 10D8 were analyzed by a high-content cell analysis system. The extent of abrin-a internalization into cells was determined by measuring the fluorescence intensity of Alexafluor-488 labeled abrin-a in the cells. There was no significant difference in the fluorescence intensity in the presence of 10D8, suggesting that 10D8 does not prevent the attachment of abrin-a to cell surface and the further internalization of abrin-a into cells (Figure 3D,E). These results indicated that the protective effects of 10D8 mainly rely on its inhibition of the enzymatic activity of abrin-a.

### 2.5. Prophylactic and Post-Exposure Treatment with 10D8 Protects against Abrin-a-Induced Lethality in Mice

**Figure 4:**
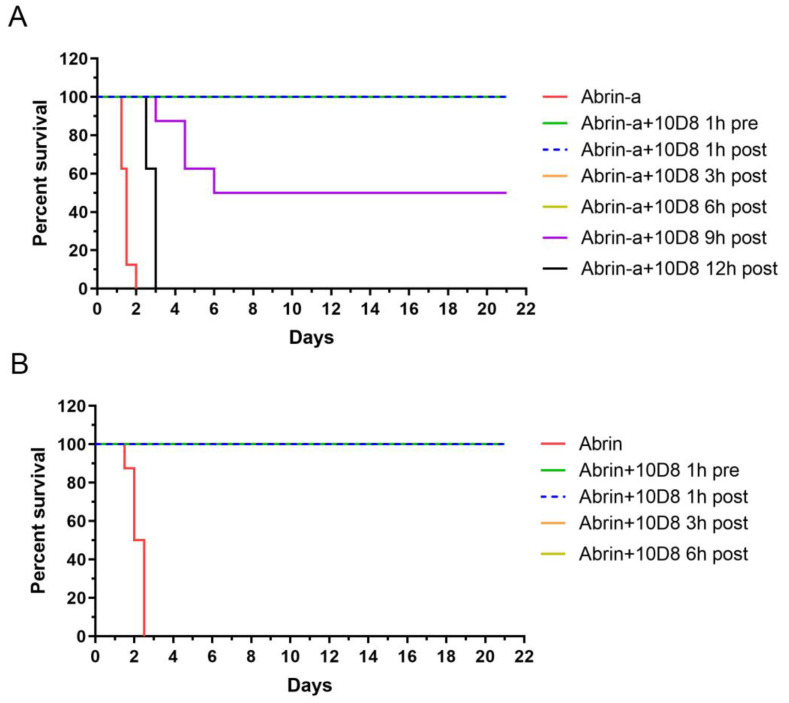
Prophylactic and post-exposure treatment with 10D8 protects against abrin-a- and abrin crude extract-induced lethality in mice. Adult BALB/c mice (eight mice per group) were injected intraperitoneally with 25 × LD_50_ of abrin-a (25 μg/kg) (**A**) or abrin crude extract (40 μg/kg) (**B**). The mAb 10D8 (3.75 mg/kg) was administered via the same route 1 h before or 1, 3, 6, 9, or 12 h after abrin-a challenge. For abrin crude extract, 10D8 (3.75 mg/kg) was administered 1 h before or 1, 3, or 6 h after challenge. The mice were monitored closely and observed for 21 days.

To assess the in vivo protective efficacy of 10D8, mice were intraperitoneally injected with 25 × LD_50_ of abrin-a or a lethal dose of abrin crude extract. The mAb 10D8 was administered via the same route 1 h before or 1, 3, 6, 9, or 12 h after abrin-a challenge. All of the mice became very ill and showed weight loss, diarrhea, drowsiness, and weakness, dying within 48 h of abrin-a intoxication without 10D8 treatment. The mAb 10D8 rescued 100% of mice from lethality via either prophylactic or 1, 3, or 6 h post-exposure treatment, and all of the mice remained healthy and lived up to 21 days (Figure 4A). In addition, 10D8 treatment at 9 h after intoxication could significantly reduce the death of intoxicated mice. However, 12 h post-intoxication treatment was ineffective, although the survival time of mice seemed prolonged. Similarly to abrin-a-intoxicated mice, mice challenged with abrin crude extract died within 60 h without 10D8 treatment. The mAb 10D8 rescued 100% of the mice from lethality via either prophylactic or 1, 3, or 6 h post-exposure treatment (Figure 4B). These results demonstrated that 10D8 treatment within 6 h after intoxication is the most effective under this lethal dosage.

### 2.6. Treatment with 10D8 Prevents Tissue Damages of Abrin-a Intoxicated Mice

**Figure 5:**
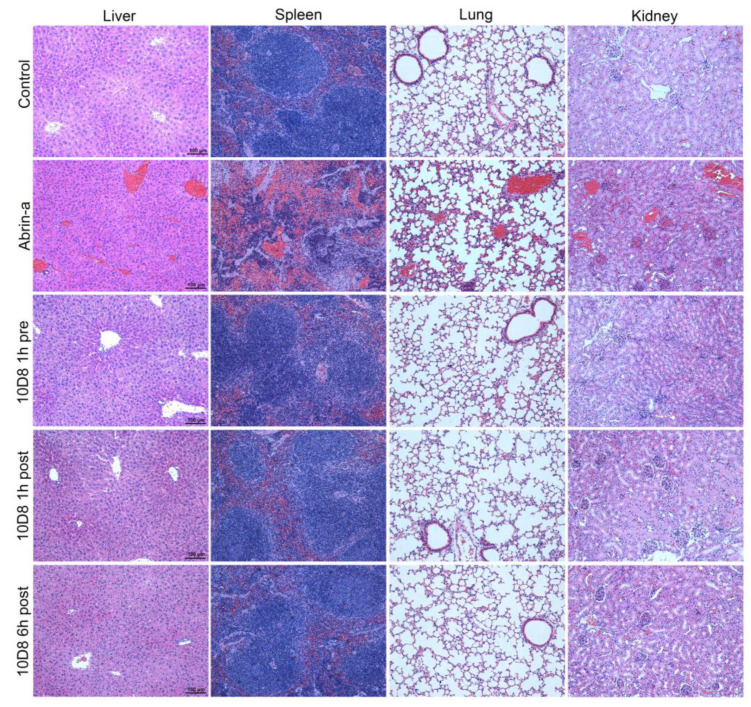
Treatment with 10D8 prevents tissue damage of abrin-a-intoxicated mice. Adult BALB/c mice were injected intraperitoneally with 25 × LD_50_ of abrin-a (25 μg/kg). The mAb 10D8 (3.75 mg/kg) was administered via the same route 1 h before or 1 or 6 h after abrin-a challenge. Livers, spleens, lungs, and kidneys were harvested and examined by hematoxylin and eosin staining 36 h after abrin-a intoxication. Scale bars: 100 μm.

Organs were harvested at 36 h after intoxication and stained with hematoxylin and eosin. Obvious hepatocyte edema and vacuolar degeneration, hepatic sinusoidal dilation, and significant congestion in the hepatic sinusoid and central vein were observed in liver tissue in the abrin-a-intoxicated group. No obvious liver damage was observed in the 10D8-treated group (Figure 5). In spleen tissue, splenic corpuscles were demolished and disappeared, red pulp widened, and white pulp atrophied in the abrin-a-intoxicated group. Meanwhile, the red pulp and white pulp areas showed obvious congestion in the abrin-a-intoxicated group. These spleen morphological changes were not observed in the 10D8-treated group (Figure 5). Abrin-a intoxication caused alveolar structure damage, alveolar wall thickening, pulmonary congestion, thrombi, and inflammatory cell infiltration in lung tissue. Similarly, 10D8 treatment prevented lung damage of abrin-a-intoxicated mice (Figure 5). The kidney tissue of mice showed severe hemorrhage, acute tubular necrosis, congested glomerulus, formation of thrombus, and inflammatory cell infiltration after abrin-a exposure. No significant kidney damage was found in the 10D8-treated group (Figure 5). On the basis of these results, it can be concluded that the immediate cause of death is multiorgan failure following abrin-a poisoning, and that 10D8 effectively protects the mice from toxin-induced tissue damage.

## 3. Discussion

Abrin is a toxin of public health concern due to its high lethality, easy availability, lack of antidote, and potential for use as a bioterrorism agent. The use of abrin in attempted murders, biothreat scenarios, and accidental and suicidal poisonings has been reported by several studies [10,22,23,24]. Despite the high toxicity, current treatment for abrin poisoning is mainly symptomatic. Currently, there are no antidotes available for managing abrin poisoning. Studies on detoxicants to rescue animals from abrin poisoning fall into two categories: (1) treatment with small molecules or antibodies post exposure; (2) prophylactic immunization by vaccine injection. Although the mechanism of toxicity of abrin at the cellular and molecular levels has been revealed up to some extent, the development of chemoprotectants is still a challenge. Antibody development for passive immunization is still recognized as the most effective therapy.

As known, it is difficult to prepare high-affinity monoclonal antibodies, especially for toxins, because the antigens with high potency may disturb the immunization process and affect high-titer antibody production in immunized animals. In the present study, inactivated abrin-a was used as the immunization antigen to establish hybridoma cell lines. Five mAbs were prepared and evaluated for the binding affinity and specificity to abrin-a. The mAb 10D8 showed the best affinity and specificity with least cross-reactivity to abrin-b and ricin (Figure 1). More importantly, it significantly inhibited cell cytotoxicity induced by abrin-a, showing a potent neutralizing activity in vitro (Figure 2). Therefore, we further explored the in vivo protection efficiency of 10D8 in a toxin challenge animal model.

So far, only rare cases have reported the protection efficacy of anti-abrin monoclonal antibodies in vivo. Antibodies were given as a prophylactic treatment in two studies, the effectiveness of which has not been demonstrated for post-exposure therapy following abrin intoxication [16,17]. In another study, mice were intranasally intoxicated with abrin (10 μg/kg, 2 × LD_50_) and treated with 100 μg of antibody at 6 h after intoxication. All of the mice without antibody treatment died by 7 days after intoxication [18]. The dose of abrin used was not high enough to induce rapid death in intoxicated mice, indicating a limited antibody post-exposure protective effect in vivo. In addition, the specificity and selectivity of the reported antibodies against abrin isoforms are completely unclear. Thus, the discovery and the development of potent anti-abrin antibodies with excellent post-exposure protective efficacy are of great importance. In our study, mice were intraperitoneally injected with 25 × LD_50_ of abrin-a, and all of the mice died within 48 h of intoxication (Figure 4A). Abrin-a intoxication also caused severe damage in the liver, spleen, lung, and kidney tissue of mice. However, prophylactic treatment or post-exposure treatment with 10D8 within 6 h could rescue 100% of mice from lethality (Figure 4A) and prevent tissue damage (Figure 5). All of the mice remained healthy and lived up to 21 days. These results suggest that 10D8 not only exhibits a neutralizing activity in cell experiments but also displays excellent in vivo pre- and post-exposure protective efficacy against abrin-a intoxication.

To address how 10D8 interacts and blocks abrin-a activity, the critical neutralizing event that confers efficient protection by the antibody was explored. As known, the abrin B chain mediates abrin binding to terminal galactose residues on glycoproteins and glycolipids expressed on the surface of cells and internalizes the cells by endocytosis through membrane vesicles. In the cytosol, the abrin A chain inactivates ribosomes by depurinating the 28S rRNA of the 60S ribosomal subunit at nucleotide position 4324, preventing the binding of rRNA to protein elongation factors and inhibiting protein synthesis [25]. To determine whether 10D8 neutralizes abrin-a through the prevention of B subunit-mediated toxin binding and internalization or abolishment of the catalytic activity of A subunit, Western blot against abrin-a was performed under reducing or nonreducing conditions. The results demonstrated that 10D8 recognizes and binds with the A chain of abrin-a but not the B chain (Figure 3A,B). Further results using the cell-free rabbit reticulocyte lysate system showed that protein synthesis is significantly inhibited by abrin-a, and that 10D8 preincubation rescues the protein synthesis of abrin-a-treated cells (Figure 3C). Next, whether 10D8 inhibits the internalization of abrin-a into cells was analyzed using a high-content cell analysis system. There was no significant difference in the fluorescence intensity in the presence of 10D8, suggesting that 10D8 does not prevent the attachment of abrin-a to the cell surface or the internalization of abrin-a into cells (Figure 3D,E). Furthermore, the results also suggested that the complex composed of abrin-a and mAb 10D8 could still be delivered intracellularly, probably through the endocytosis mediated by B chain. The above results indicated that the protective effects of 10D8 mainly rely on its inhibition of the enzymatic activity of abrin-a, in line with the previous reported antibody D6F10 [26]. However, as shown by the results, 10D8 only partially inhibits the enzymatic activity of abrin-a and, consequently, partially reduces protein synthesis inhibition (Figure 3C), while completely blocking the toxicity of abrin-a in cells and in mice. We suppose that this may be due to the different conditions between cell-free systems and cells. Firstly, the toxin may be more potent in inhibiting the enzymatic activity under a simple environment such as the cell-free system. In addition, toxins undergo much more complex processes such as attachment, internalization, transportation, and degradation in cells, which may affect the inhibiting activity of the antibody. Lastly, protein synthesis ceased after abrin-a intoxication, finally leading to cell death. Treatment with 10D8 recovered a large proportion of protein synthesis, which was potentially enough to sustain the normal function of cells and eventually protected cells and mice from death.

Taken together, we prepared, identified, and acquired a high-affinity neutralizing monoclonal antibody 10D8 with a potent pre- and post-exposure protective effect against the cytotoxicity and animal toxicity of abrin-a. The high affinity, good specificity, and potent antitoxic efficiency of 10D8 make it an excellent candidate for therapeutic antibodies against abrin. Further research on the antitoxic mechanism of mAb 10D8 against abrin intoxication will be conducted in the future, which may facilitate the application of mAb 10D8 in humans.

## 4. Materials and Methods

### 4.1. Materials

Abrin crude extract containing abrin isoforms and AAG was prepared from *Abrus precatorious* seeds from Yunnan province, China. Abrin-a, abrin-b, AAG, and ricin were prepared and purified in-house with milligram yield as described previously [27,28]. The purity of abrin-a, abrin-b, and AAG was estimated to be higher than 95% (Figure 3A and Supplementary Material Figure S2) [19,29]. RCA120 was purchased from Sigma-Aldrich. All toxins were handled by trained personnel with protective gloves and goggles in a well-ventilated fume hood. Toxin-containing solutions and consumables were totally decontaminated with sodium hydroxide and inactivated by autoclaving.

### 4.2. Ethics Statement

This study was carried out in strict accordance with the approved guidelines. All animal procedures were reviewed and approved by the Institutional Animal Care and Use Committee of Laboratory Animal Center, Academy of Military Medical Sciences (AMMS), China (Assurance Number: IACUC-DWZX-2021-593; date of approval: 4 March 2021).

### 4.3. Antigen Inactivation for Immunization

Purified abrin-a protein dissolved in PBS at a concentration of 2 mg/mL was dialyzed using a Slide-A-Lyzer dialysis cassette (molecular weight cutoff of 10,000) against 4% paraformaldehyde solution at 47 °C for 16–18 h. Then, the cassette was transferred to PBS solution (pH 7.4) to remove the remaining paraformaldehyde through dialysis, and the procedure was repeated every 12 h for a total dialysis time of 72 h. At last, the abrin-a solution in the cassette was pipetted and collected into a new tube. The concentration of purified inactivated abrin-a protein was determined by BCA assay.

### 4.4. Preparation of Anti-Abrin-a mAbs

Six week old BALB/c mice were immunized intraperitoneally with 33 μg of inactivated abrin-a mixed with Freund’s complete adjuvant (Sigma-Aldrich, St. Louis, MO, USA) four times at 2 week intervals. Hybridomas were produced by fusion of spleen cells from the immunized mice with myeloma cells 4 days after the last immunization, according to a published procedure [30]. Briefly, splenocytes were fused with NS-1 myeloma cells to produce fused hybridoma cells which were selectively cultured in RPMI 1640 medium supplemented with 10% fetal calf serum, hypoxanthine, aminopterin, and thymidine (Sigma-Aldrich, St. Louis, MO, USA). Hybridoma supernatants were screened for the presence of mAbs against abrin by indirect ELISA using abrin as the coated antigen. Positive hybridoma cells were cloned by limiting dilution, and the positive hybridomas were cloned at least twice by limiting dilution. Antibody subclasses were determined by rapid isotyping cassettes (Thermo Fisher Scientific, Rockford, IL, USA). Monoclonal antibodies were purified by protein G column chromatography (Amersham-Pharmacia, Uppsala, Sweden) according to the manufacturer’s instructions. The purified mAbs were labeled with HRP (Sigma-Aldrich, St. Louis, MO, USA) using the periodate method. The antigen-binding abilities of the mAbs were previously determined using the method developed by Beatty et al. [31], and affinity constants were also further determined using the SPR method established in our lab [19].

### 4.5. Indirect ELISA

Indirect ELISAs were employed to screen the established hybridomas. Briefly, microwell plates were coated with 100 µL of 5 µg/mL abrin protein in 0.05 M bicarbonate buffer (pH 9.6) at 4 °C overnight and blocked with 300 µL of 1% (*m*/*v*) BSA for 2 h at 37 °C. After the blocking steps, 100 µL of supernatant from the hybridoma cultures serially diluted in PBST containing 0.5% BSA was added to the wells to incubate for 1 h at room temperature. After the wells were washed three times with PBST, bound antibodies were detected with horseradish peroxidase (HRP)-labeled goat anti-mouse IgG antibody (Abcam, Cambridge, UK). Following washing, the colorimetric reaction was measured using 3,3′,5,5′-tetramethylbenzidine (TMB) peroxidase substrate after a 10 min development period. The reaction was then terminated with 2 M H_2_SO_4_ (Sigma-Aldrich, St. Louis, MO, USA), and the absorbance at 450 nm was measured using a microplate reader (Infinite M1000 pro, TECAN, Männedorf, Switzerland).

### 4.6. ELISA for Cross-Reactivity Evaluation of mAbs

Microplates were coated with 100 µL 10 μg/mL of either abrin-a, abrin-b, AAG, ricin, or RCA120 in 0.05 M bicarbonate buffer (pH 9.6) at 4 °C overnight and blocked with 300 µL of 1% (*m*/*v*) BSA for 2 h at 37 °C. Then, 100 µL of 10 μg/mL screened mAbs were added and incubated for 1 h at 37 °C. Wells were washed with PBST, before adding 100 µL of 1:1000 dilution of HRP-labeled anti-mouse antibody and then incubating for 40 min at 37 °C. Wells were washed with PBST, before adding 100 µL of TMB substrate solution and incubating for 10 min. The reaction was terminated by 50 µL of 2 M H_2_SO_4_. Absorbance at 450 nm was measured using a microplate reader (Infinite M1000 pro, TECAN, Männedorf, Switzerland).

### 4.7. Cell Culture

HeLa cells and NS-1 myeloma cells were cultured in RPMI-1640 medium (Gibco, Grand Island, NY, USA) supplemented with 10% fetal bovine serum (Gibco, Grand Island, NY, USA), 100 units/mL penicillin, and 100 μg/mL streptomycin (Gibco, Grand Island, NY, USA), and incubated at 37 °C in the presence of 5% CO_2_.

### 4.8. Cell Cytotoxicity Assay

HeLa cells were seeded in 96-well cell culture plates at a density of 1 × 10^4^ cells/well. For the cell death study, HeLa cells were pretreated with 50 μM Z-VAD-FMK (Selleck, Shanghai, China), 50 μM Nec-1 (Selleck, Shanghai, China), or 100 nM wortmannin (Selleck, Shanghai, China) 1 h prior to abrin-a intoxication. Then, abrin-a was diluted with serum-free RPMI-1640 medium at 10 or 100 ng/mL and added into each well. Toxins were diluted with serum-free RPMI-1640 medium at various concentrations and preincubated with or without 10 μg/mL monoclonal antibodies at 37 °C for 1 h. Alternatively, antibody 10D8 was added to cells 1, 3, or 6 h after intoxication. Cells were incubated at 37 °C for 24 h. A10 μL aliquot of CCK-8 reagent (Beyotime, Shanghai, China) was added into each well and incubated at 37 °C for 1 h. Absorbance at 450 nm was measured using a microplate reader (Infinite M1000 pro, TECAN, Männedorf, Switzerland). The survival rate of cells was calculated as follows: survival rate % = (A_450 sample_/A_450 control_) × 100%.

### 4.9. Cell-Free Luciferase Assay for Analyzing Protein Synthesis

Abrin-a (10 ng/mL) alone or along with 10D8 (10 μg/mL) was added to TNT^®^ T7 coupled reticulocyte lysate systems (Promega, Madison, WI, USA) containing total amino acids, RNase inhibitor, and luciferase mRNA and incubated for 1.5 h at 37 °C. After the incubation, 1 μL of the reaction mixture containing synthesized luciferase was mixed with 50 μL of luciferase assay reagent pre-equilibrated to room temperature, and the extent of the synthesized luciferase was evaluated by measuring the luminescence.

### 4.10. Immunofluorescence Assay

HeLa cells were seeded in a 96-well black cell culture plate at a density of 1 × 10^4^ cells/well. The cells were treated with abrin-a (100 ng/mL) or abrin-a preincubated with 10D8 (20 μg/mL) for 3 h at 37 °C. The cells were washed three times with PBS and fixed with 4% paraformaldehyde. The fixed cells were incubated with 0.1% Triton X-100 for 30 min for penetration and blocked for 30 min with 5% BSA at room temperature. The cells were treated with 10D8 for 2 h at 37 °C and washed three times, before incubating with Alexa-488 labeled anti-mouse IgG (Invitrogen, Carlsbad, CA, USA) for 1 h and washing three times. The nuclei were stained with Hoechst-33342. The plate was visualized and analyzed using a high-content cell analysis system (Opera Phenix, PerkinElmer, Waltham, MA, USA).

### 4.11. SDS-PAGE

Firstly, 4 μg of abrin-a, abrin-b, and AAG and 20 μg of abrin crude extract were loaded and separated on 10% sodium dodecyl sulfate polyacrylamide gel electrophoresis under reducing and nonreducing conditions, stained with Coomassie Blue solution (Beyotime, Shanghai, China).

### 4.12. Western Blot

Firstly, 100 ng of abrin-a was loaded and separated on 10% SDS polyacrylamide gels under reducing or nonreducing conditions, before transferring onto a polyvinylidene difluoride (PVDF) membrane. After blocking with 5% BSA, the membranes were incubated with 10D8 antibody diluted to 1:1000 and HRP-labeled anti-mouse secondary antibody (Abcam, Cambridge, UK) diluted to 1:5000. The immunostaining signal was visualized using ECL Plus reagent (Applygen, Beijing, China) and imaged with FluorChem E (ProteinSimple, San Jose, CA, USA).

### 4.13. Mice Intoxication Model

Adult BALB/c mice (eight mice per group) were injected intraperitoneally with 25 × LD_50_ of abrin-a (25 μg/kg) [9,32,33] or a lethal dose of abrin crude extract (40 μg/kg). The mAb 10D8 (3.75 mg/kg) was administered via the same route 1 h before or 1, 3, 6, 9, or 12 h after abrin-a challenge. For abrin crude extract, 10D8 (3.75 mg/kg) was administered 1 h before or 1, 3, or 6 h after challenge. The mice were monitored closely and observed for up to 21 days after injection. Organs were harvested at 36 h after intoxication. The morphologies of the heart, liver, spleen, lung, and kidney of mice were examined by observing hematoxylin and eosin-stained sections of the respective tissues (BX51, OLYMPUS, Tokyo, Japan).

### 4.14. Statistical Analysis

Data were expressed as the means ± standard deviation (SD) of three independent experiments. Statistical analyses were performed using one-way analysis of variance (ANOVA) followed by Tukey’s multiple comparisons test with GraphPad Prism 8 software (* *p* < 0.05, ** *p* < 0.01, *** *p* < 0.001).

## Data Availability

Data is contained within the article or supplementary material.

## Supplementary Materials

The following supporting information can be downloaded at: https://www.mdpi.com/article/10.3390/toxins14030164/s1: Figure S1. The subclasses of mAbs determined by rapid isotyping cassettes represented by 10D8. Figure S2. Mascot search results showing library match to abrin-a, abrin-b, AAG and abrin crude extract. Table S1.The affinity and kinetics constants of mAbs against abrin-a and isotypes.
