# Supplementary material for: Neutralizing Monoclonal Antibody, mAb 10D8, Is an Effective Detoxicant against Abrin-a Both In Vitro and In Vivo
Source: Toxins (Basel). 2022 Feb 23;14(3):164. doi: 10.3390/toxins14030164 (PMC8955035; doi:10.3390/toxins14030164)
Supplement: Supplementary file 1 [file toxins-14-00164-s001.zip › toxins-1594110-supplementary.pdf]

toxins

Supplementary Materials: Neutralizing Monoclonal Antibody, mAb 10D8, Is an Effective Detoxicant against Abrin-a Both In Vitro and In Vivo

Zhi Li, Hua Xu, Bo Ma, Li Luo, Lei Guo, Pingping Zhang, Yong Zhao, Lili Wang and Jianwei Xie

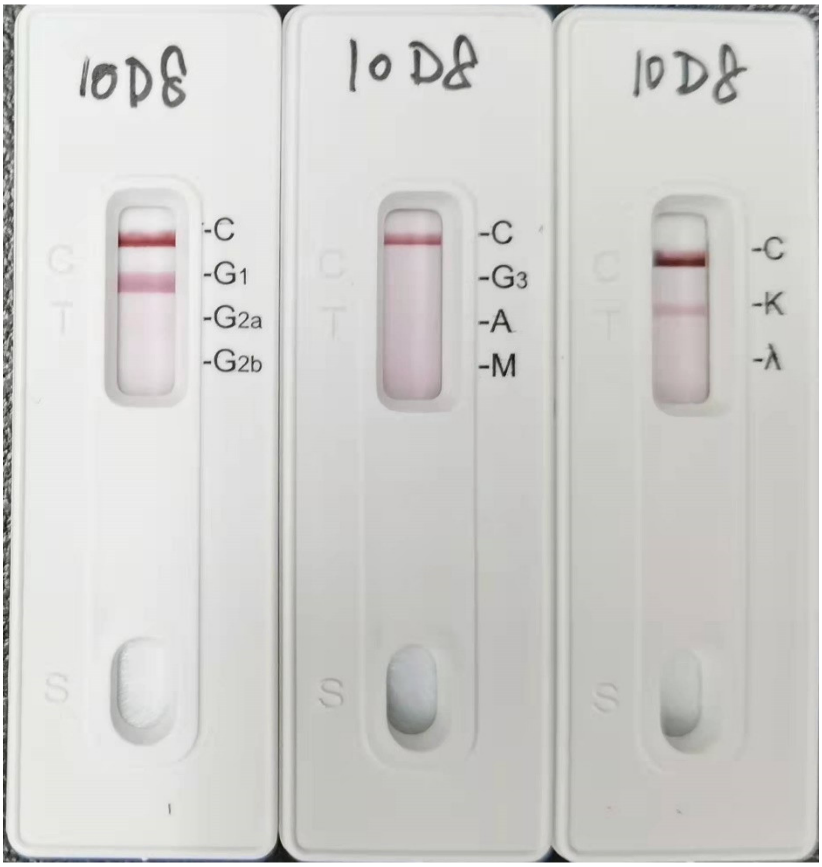

Figure S1. The subclasses of mAbs were by rapid isotyping cassettes represented by 10D8.

Table S1. The affinity and kinetics constants of mAbs against abrin-a [19] and isotypes.

	ka ($M^{-1}s^{-1}$)	kd (s^{-1})	K_D (pM)	isotype
10D8	(13.1 ± 0.8)×10^6	(5.1 ± 0.3)×10^{-5}	4.9 ± 0.5	IgG1 Kappa Chain
10C9	(3.0 ± 0.1)×10^6	(0.6 ± 0.1)×10^{-5}	2.2 ± 0.1	IgG1 Kappa Chain
5A10	(1.4 ± 0.6)×10^7	(1.6 ± 0.004)×10^{-5}	1.4 ± 0.6	IgG1 Kappa Chain
5G7	(1.3 ± 0.1)×10^6	(3.9 ± 0.9)×10^{-5}	32 ± 1.0	IgG1 Kappa Chain

The affinity and kinetics constants of mAbs against abrin-a were determined by the SPR method established in our lab [19]. The subclasses of mAbs were determined by rapid isotyping cassettes (Thermo Fisher Scientific, Rockford, IL, USA).

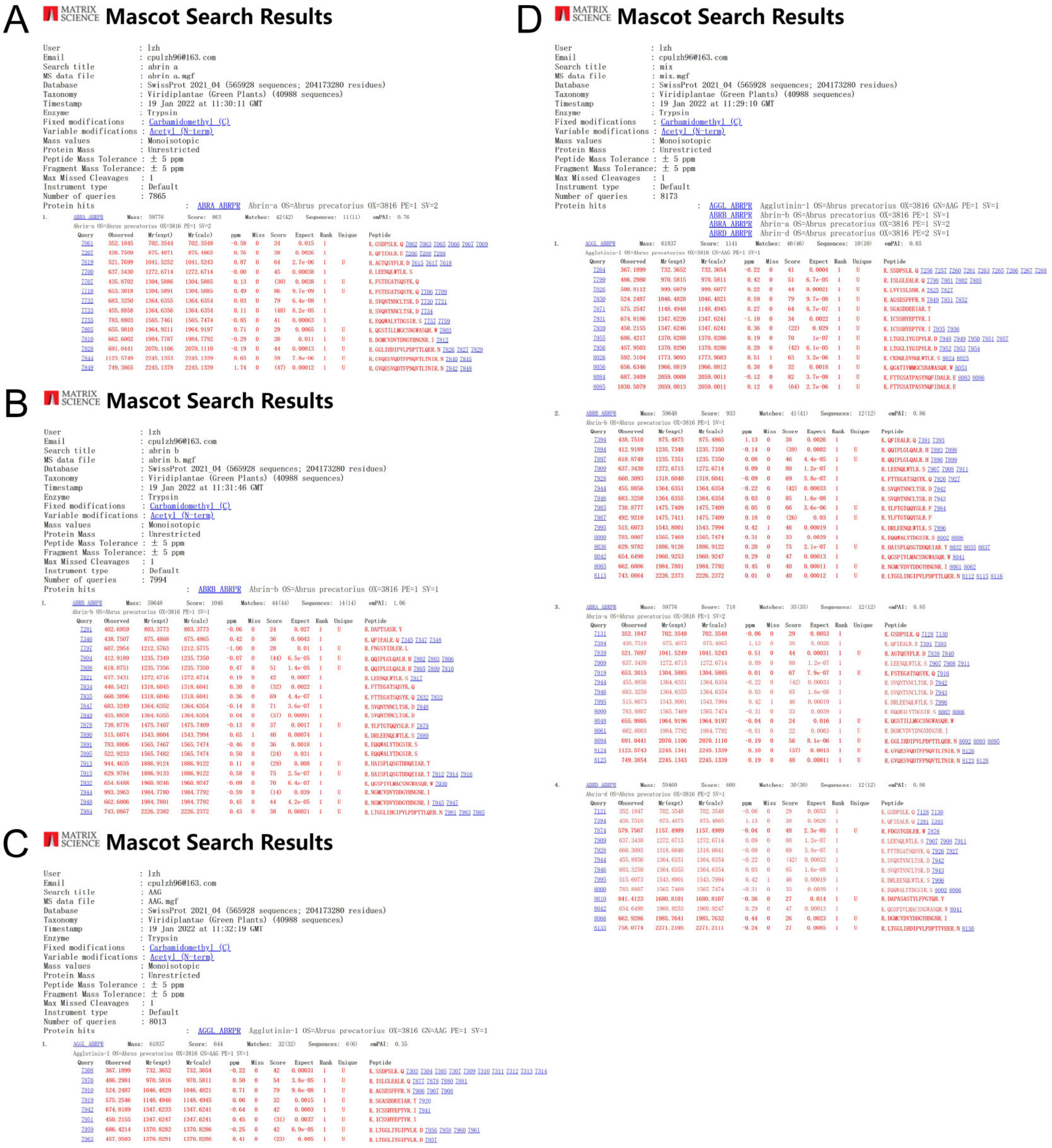

Figure S2. Mascot search results showing library match to abrin-a (**A**), abrin-b (**B**), AAG (**C**) and abrin crude extract (**D**). Abrin-a (5 µg), abrin-b (5 µg), AAG (5 µg) and abrin crude extract (10 µg) were reduced with dithiothreitol (Thermo Fisher Scientific, Rockford, IL, USA), alkylated by iodoacetamide (Thermo Fisher Scientific, Rockford, IL, USA), digested by trypsin (Thermo Fisher Scientific, Rockford, IL, USA) and analyzed by LC-MS/HRMS. Library searches were performed with Mascot using raw MS/HRMS data against an online database.

Reference

19. Luo, L.; Yang, J.; Li, Z.; Xu, H.; Guo, L.; Wang, L.; Wang, Y.; Luo, L.; Wang, J.; Zhang, P.; et al. Label-free differentiation and quantification of ricin, abrin from their agglutinin biotoxins by surface plasmon resonance. *Talanta* **2022**, *238*, 122860, doi:10.1016/j.talanta.2021.122860.
